# 

**Published:** 1910-05-14

**Authors:** 


### THE QUEEN-MOTHER'S THANKS TO HER PEOPLE.

Buckingham Palace, May 10.

From the depth of my poor broken heart I wish to express to the whole Nation and our kind People we love so well my deep-felt thanks for all their touching sympathy in my overwhelming sorrow and unspeakable anguish.

Not alone have I lost everything in him, my beloved Husband, but the Nation, too, has suffered an irreparable loss by their best friend, Father, and Sovereign thus suddenly called away.

May God give us all His divine help to bear this heaviest of crosses which He has seen fit to lay upon us—"His Will be done." Give me a thought in your prayers which will comfort and sustain me in all I still have to go through.

Let me take this opportunity of expressing my heartfelt thanks for all the touching letters and tokens of sympathy I have received from all classes, high and low, rich and poor, which are so numerous that I fear it will be impossible for me ever to thank everybody individually.

I confide my dear Son into your care, who I know will follow in his dear Father's footsteps, begging you to show him the same loyalty and devotion you showed his dear Father.

I know that both my dear Son and Daughter-in-law will do their utmost to merit and keep it.

Alexandra.

### A DAY OF MOURNING.

An official proclamation was published in last Tuesday's *London Gazette* appointing Friday, the 20th inst., as a day of general mourning in accordance with statute.

The Earl Marshal promulgated, in a supplement published the same evening, the following General Order:—

In pursuance of an Order of his Majesty in Council, dated the 10th day of May, 1910, these are to give public notice that it is expected that all persons upon the present occasion of the death of his late Majesty of Blessed and Glorious Memory, do put themselves into decent mourning, to begin on Thursday, the 12th day of this instant May.

### Jottings.

T.M. King George and Queen Mary have become patrons of the Italian Hospital, Queen Square.

Lord Howard de Walden will preside at a festival dinner in November next to celebrate the 125th anniversary of the St. Marylebone General Dispensary (commonly called the "Welbeck Dispensary"). Opportunity will be taken to relieve the charity of an old-standing rebuilding debt. The last dinner was held at the centenary celebration twenty-five years ago, when the late Alderman Sir Richard Carden presided.

At a meeting of the Leeds Workpeople's Hospital Fund, on April 27, on the motion of the chairman, seconded by the Rev. S. Manson, and supported by Mr. J. Tomlinson, a vote was accorded to Lord Airedale heartily thanking him for his generous and wholly unsolicited gift of £100, as a special donation for the Workpeople's Convalescent Home at Horsforth, in commemoration of his opening the enlarged Home in 1907.

The Council of the Pharmaceutical Society of Great Britain has awarded the Pereira medal to Mr. Walter Ryley Pratt, a student of the Royal School of Pharmacy. The Pereira medal was founded in 1874 in memory of the late Dr. Jonathan Pereira, M.D., F.R.S., a former Professor of Materia Medica to the Pharmaceutical Society. The Society's silver medal was awarded to Mr. Howard Vincent Potter, of Hastings.

A Convalescent Home for navvies is to be opened—the site, we believe, has not yet been decided on—as soon as the necessary money has been subscribed. The home will receive men during the convalescent period. Men in receipt of compensation will not be eligible. Over £400 is required to start, and Colonel Charles Dennys, "Ardtona," Budleigh Salterton, Devon, will be glad to receive donations. Mr. F. Moulton-Barrett, Woodbury, R.S.O., Exeter, will gladly give all information.

A grand hospital matinée in aid of the funds of the Leicester Infirmary is to take place on Thursday, May 19. Mr. Oswald Stoll has generously granted the free use of the Palace Theatre and the services of the artists and orchestra for the occasion, and it is hoped a large sum will accrue to the Infirmary. An enthusiastic committee has been appointed with the Mayor of Leicester as President, Mr. H. H. Wildt as Chairman, Messrs. J. H. Ward and C. Finch Hatton as Hon. Secretaries, and Mr. Arthur Baum as Treasurer.

We take the following letter from the *Times*, written by the editor of the *Poultry World*, address 154 Fleet Street: "About this time last year we succeeded, with your friendly help, in obtaining nearly 20,000 eggs, all of which were immediately distributed amongst the various deserving metropolitan hospitals. In the month of May eggs are most plentiful, and consequently keepers of laying fowls will be well able to spare at least one dozen new laids from their surplus. These should be sent to me between the dates of May 18 and May 26, carefully packed and carriage paid. All boxes used will be returned on request. Special labels for despatching eggs will be sent on application. The co-operation of the Hospital Saturday Fund has again been secured in the allocation of the gifts, and it is earnestly hoped that all your readers who can send eggs will do so. They are sorely needed. All packages containing eggs will be sent direct to the hospitals without being opened, so it is necessary that any covering letter should be despatched separately. It is also advisable to state the number of eggs contained in each package on the outside of such consignment. Every gift of eggs will be acknowledged."

### Gifts and Bequests.

The Skinners' Company has sent ten guineas to the British Lying-in Hospital, Endell Street.

The Merchant Taylors' Company has given twenty guineas to Queen Charlotte's Hospital.

Mr. Siegmund Hermann Epstein has left £100 to King Edward's Hospital Fund for London.

The Chelsea Hospital for Women has received a grant of £31 10s. from the Merchant Taylors' Company.

Mr. John William Smith has left, on the death of his wife, £450 to the Western General Dispensary.

The Royal Westminster Ophthalmic Hospital has received twenty guineas from the Merchant Taylors' Company.

Miss Florence Ethel Hudson, of Sale, Cheshire, has left £2,000 to St. Mary's Hospital for Women and Children, Manchester.

The City Corporation has given £100 and the Glovers' Company five guineas to the Royal Hospital for Incurables, Putney Heath.

Mr. Howard Morley has sent a donation of £1,000 to University College Hospital, in response to the special appeal for £10,000 before June 1.

The North London Nursing Association for Providing Trained Nurses for the Sick Poor has received a grant of £10 15s. from the Clothworkers' Company.

Leicester Infirmary has received from the late Miss Elizabeth Coleman, of Leicester, £20 to the Infirmary and £20 to the Children's Hospital in connection with the Infirmary.

The Grocers' Company has made a grant of £100 to the funds of the Royal Hospital for Incurables, Putney Heath.

The Chelsea Hospital for Women has received a grant of £31 10s. from the Merchant Taylors' Company.

Mr. Thomas Taylor, licensed victualler, of Derby, has left £1,000 to the Derbyshire Royal Infirmary, £500 to the Derby Hospital for Sick Children, and £300 to the Derbyshire Infirmary Convalescent Home at Holbrook.

University College Hospital acknowledges the receipt of the following contributions in response to special appeal for a sum of £10,000 before June 1, 1910: New Annual Subscription: The Baroness Eckardstein, £20. Donations: Howard Morley, Esq., £1,000; the Company of Mercers, £210; Claude G. Montefiore, Esq., per H. Lucas, Esq., £200; Sir Thomas Barlow, Bart., F.R.C.P., £100; Lady Barlow, £100; Mrs. Mirrielees, £100; F. Nettlefold, Esq., per R. Worsley, Esq., £50; C. F. Pearson, Esq., £30; R. Worsley, Esq., £25; "A Friend," per H. Lucas, Esq., £25; Messrs. Coutts and Co., £21, and many others.

### Appointments.

Mr. Somerville Hastings, M.S., F.R.C.S., has been appointed Aural Surgeon to the Middlesex Hospital.

Mr. A. J. Fuller, B.A., M.R.C.S., L.R.C.P., has been appointed house physician to the Leicester Infirmary. Mr. Fuller was previously house surgeon to Mr. Harrison Cripps and Mr. Waring at St. Bartholomew's Hospital for twelve months.

Mr. Alexander Wilson, M.B., Ch.B. (Aberdeen), has been appointed Demonstrator in Anatomy to the University of Sheffield. Mr. H. K. Stephenson, J.P., has been appointed representative of the University of Sheffield on the West Riding County Association.

Sir Francis Lovell, the Dean of the London School of Tropical Medicine, who left England last November for the West Indies, has just returned. His object was to interest some of the Colonies in the work of the school, and to obtain subsidies from them in aid of research work in tropical disease. As the result of his efforts each Colony has undertaken to make a grant to the School of Tropical Medicine. The total amount expected will be nearly £500 a year for the next five years. The London School of Tropical Medicine since its establishment ten years ago has trained 1,100 postgraduates in the study and investigation of tropical diseases. Sir Francis Lovell saw many cases of pellagra while in Barbados, where Dr. Louis W. Sambon, of the London School of Tropical Medicine, is conducting a scientific investigation into the causes of the disease.

Mr. E. Bainbridge, Mr. W. Blake Burdekin, Mr. George Denton, Mr. R. W. Fowler, Mr. W. S. Laycock, Mr. T. P. Lockwood, Mr. W. A. Matthews, Mr. J. G. Ronksley, Mr. T. W. Sorby, Mr. Charles E. Vickers, Mr J. D. Webster, and the Rev. J. E. Jump have been elected to the Committee of the Sheffield Children's Hospital.

The Managing Committee of the Children's Hospital, Harcourt Street, Dublin, has been elected as follows for the coming year : Sir Henry L. Harty, Bart.; Sir Lambert H. Ormsby, M.D.; Sir Charles A. Cameron, C.B.; Mr. H. Lindsay-Fitzpatrick, D.L.; Mr. A. H. Ormsby-Hamilton, D.L.; Mr. Stanley Cochrane; Lieut.-Colonel J. H. Dopping, J.P.; Lieut.-Colonel Arthur Hartley, J.P.; Lieut.-Colonel A. H. L'Estrange; Lieut.-Colonel Walter P. Carson; Mr. Marmaduke Backhouse, C.E.; Mr. Robert Gidney; Mr. Henry Hunt, B.L.; Dr. Wm. J. Gibson, M.D., J.P.; Mr. William Ireland,, J.P., T.C.; Dr. R. S. Wayland; Mr. Arthur Raymond Pelly; Lieut.-Colonel W. T. Johnston; Lieut.-Colonel F. H. Macnamara, while Alderman J. Delahunt and Councillor M. Maher are Dublin Corporation representatives.

THE

### GRADUATES' MEDICAL SCHOOLS.

DIARY FOR THE WEEK, MAY 16 to MAY 21.

**LONDON SCHOOL OF CLINICAL MEDICINE,** Seamen's Hospital, Greenwich, S.E.

At 4 p.m.—**May 19,** Dr. T. Lister, **Infant Feeding.**

**NORTH-EAST LONDON POST-GRADUATE COLLEGE,** Prince of Wales's General Hospital, Tottenham, N.

At 4.30 p.m.—**May 17,** Dr. R. Murray Leslie, **Irregularity of the Heart, its Causes and Significance.**
**May 19,** Mr. Walter Edmunds, **Surgical Tuberculosis.**

**THE BROMPTON HOSPITAL FOR CONSUMPTION AND DISEASES OF THE CHEST,** Fulham Road, S.W.

At 4 p.m.—**May 18,** Mr. Godlee, **Foreign Bodies in the Air Passages.**

**THE POST-GRADUATE COLLEGE,** West London Hospital, Hammersmith, W.

At 10 a.m.—**May 19,** Demonstration by Surgical Registrar.
10.30 a.m.—**May 17,** Demonstrations of Minor Operations.
12.15 a.m.—**May 18,** Dr. Grainger Stewart, **Practical Medicine.**
5 p.m.—**May 17,** Dr. Seymour Taylor, **Typhoid.**
5 p.m.—**May 18,** Dr. Morton, **X-Rays Examination of the Digestive System** (Illustrated Lectures).
5 p.m.—**May 19,** Mr. Armour, **Operations for Hernia.**

**MEDICAL GRADUATES' COLLEGE AND POLYCLINIC,** 22 Chenies Street, W.C.

**May 16.**—Whit Monday.
At 4 p.m.—**May 17,** Dr. Essex Wynter, **Clinique (medical).**
5.15 p.m.—**May 17,** Dr. G. C. Cathcart, **How to Treat Sore Throat.**
4 p.m.—**May 18,** Mr. T. P. Legg, **Clinique** (surgical).
5.15 p.m.—**May 18,** Dr. L. H. Pegler, **Headaches in Relation to Obstructions in the Nasal Passages.**
4 p.m.—**May 19,** Sir Jonathan Hutchinson, **Museum Demonstration.**
5.15 p.m.—**May 19,** Mr. P. Lockhart Mummery, **The Treatment of Internal Piles.**
**May 20.**—College closed.

The retiring trustees of the North Charitable Infirmary and County and City of Cork General Hospital have been re-elected as follows: The Most Rev. Dr. O'Callaghan; Mr. Albert J. Cagney; Mr. Patrick Casey, T.C.; Mr. A. M. Cole, J.P.; Dr. D. D. Donovan, M.D.; Mr. D. H. Forde, the Rev. W. J. Galway, LL.D.; Mr. Benjamin Haughton; Sir Daniel Hegarty, J.P.; Mr. Maurice Healy, M.P.; Mr. Patrick Harding; Mr. Louis J. Murphy; the Rev. Martin Murphy, Adm.; Mr. John C. Newsom, J.P.; Mr. Stephen Perry, J.P.

THE HOSPITAL. May 14, 1910.

*Name* ..............................

*Address* ..............................

..............................

This Coupon must accompany manuscript or contributions intended for The Hospital.

